# RNA-based qPCR as a tool to quantify and to characterize dual-species biofilms

**DOI:** 10.1038/s41598-019-50094-3

**Published:** 2019-09-20

**Authors:** Andreia Patrícia Magalhães, Ângela França, Maria Olívia Pereira, Nuno Cerca

**Affiliations:** Centre of Biological Engineering, LIBRO – Laboratório de Investigação em Biofilmes Rosário Oliveira, University of Minho, Campus de Gualtar, 4710-057 Braga, Portugal

**Keywords:** Biofilms, Microbial ecology

## Abstract

While considerable research has focused on studying individual-species, we now face the challenge of determining how interspecies interactions alter bacterial behaviours and pathogenesis. *Pseudomonas aeruginosa* and *Staphylococcus aureus* are often found to co-infect cystic-fibrosis patients. Curiously, their interaction is reported as competitive under laboratory conditions. Selecting appropriate methodologies is therefore critical to analyse multi-species communities. Herein, we demonstrated the major biases associated with qPCR quantification of bacterial populations and optimized a RNA-based qPCR able not only to quantify but also to characterize microbial interactions within dual-species biofilms composed by *P*. *aeruginosa* and *S*. *aureus*, as assessed by gene expression quantification. qPCR quantification was compared with flow-cytometry and culture-based quantification. Discrepancies between culture independent and culture dependent methods could be the result of the presence of viable but not-cultivable bacteria within the biofilm. Fluorescence microscopy confirmed this. A higher sensitivity to detect viable cells further highlights the potentialities of qPCR approach to quantify biofilm communities. By using bacterial RNA and an exogenous mRNA control, it was also possible to characterize bacterial transcriptomic profile, being this a major advantage of this method.

## Introduction

The recognition that biofilm communities, which typically comprise multiple species^1^, with a spatiotemporal heterogeneous chemical, physiological and genetic composition^2^ poses a serious healthcare concern regarding the synergies that arise from the co-infecting species^3,4^. Hence, selecting appropriate methodologies that provides reliable quantitative measures of individual populations within the polymicrobial biofilms is crucial to predict the behaviour and dynamic of these communities.

Culture-based methods have long been used to detect microbial pathogens; however, since these methodologies require the growth of the cultures, it takes a long period of time to obtain results. Furthermore, culturing is not always an option since some bacterial species are difficult to growth in the laboratory^5,6^ and many species develop a viable but non-cultivable state^7–9^. Other limitations are the presence of cell-aggregates that strongly influence the outcome of bacterial culture quantification^10,11^. Alternatively, culture-independent assessments of microbial communities, particularly through quantitative real-time PCR (qPCR) or sequencing, have allowed a rapid screening and/or quantification of the specific microorganisms within the consortia^12–16^. The most frequently used qPCR method is based in the amplification of gDNA molecules. Unfortunately, DNA-based methodologies cannot unequivocally differentiate between live (including viable but non-cultivable cells (VBNC)) and dead cells^17–20^ and, therefore, are likely to overestimate the microbial communities^19^. Other specialized methods, such as propidium-monoazide (PMA)-qPCR^21^, are becoming more popular as the function and organization of the entire community depends on which members are alive or dead^22^. Nevertheless, PMA-based approaches have reportedly important limitations, including changes in cell membrane permeability (influencing the uptake of PMA dye) and false-positive signals^23^.

A less explored alternative is the utilization of RNA-based qPCR approaches for the detection of live members of a polymicrobial consortia. This principle was recent demonstrated by several independent studies which demonstrated that RNA-based quantification, was superior to DNA-based methods, in detecting live bacterial cells, providing a more relevant insight into the microbial community composition^24,25^. Of utmost importance, RNA-based qPCR approach has the significant potential of performing not only a quantification analysis, but also addressing specific microbial interactions, as detected by the expression of key virulence genes of interest.

In fact, infections caused by the presence of multispecies are often more virulent or recalcitrant to treatment than those caused by each independent species^26^. However, relatively little is known about the interspecies interactions that modulate the community dynamics in terms of composition and virulence; ultimately contributing to increased infection severity and chronicity^27^.

*P. aeruginosa* and *S. aureus* are versatile bacterial pathogens and common etiological agents in several polymicrobial infections, including wounds, otitis media and oral infections, and CF lung disease^3^. Of particular interest is the ecological interactions between *P*. *aeruginosa* and *S*. *aureus*, since microbial communities containing both of these pathogens can display enhanced virulence^27^. Despite routine administration of antibiotics, these infections are often highly resilient and tolerant to treatment^28^. This might explain why despite aggressive antibiotics treatment, patients with CF will eventually succumb to the chronic persistent infections^29,30^. Robust measures of these community changes (composition and/or abundance) are therefore urgent, since misinterpretation of these communities can impair antimicrobial treatment^31–34^.

Herein, the objective of this study was to evaluate an optimal RNA-based qPCR method to investigate the community dynamics and microbial interactions between *P*. *aeruginosa* and *S.* *aureus*. Thus, microbial composition of 24- and 48-h-old dual-species biofilms was assessed by RNA-based qPCR as well as changes in the transcriptomic profile of key virulence-related genes. The qPCR bacterial quantification was first fully optimized to polymicrobial samples and further compared with conventional culture and flow-cytometry techniques.

## Results and Discussion

### Quantification of dual-species biofilm communities: plate count *versus* flow cytometry

**Figure 1:**
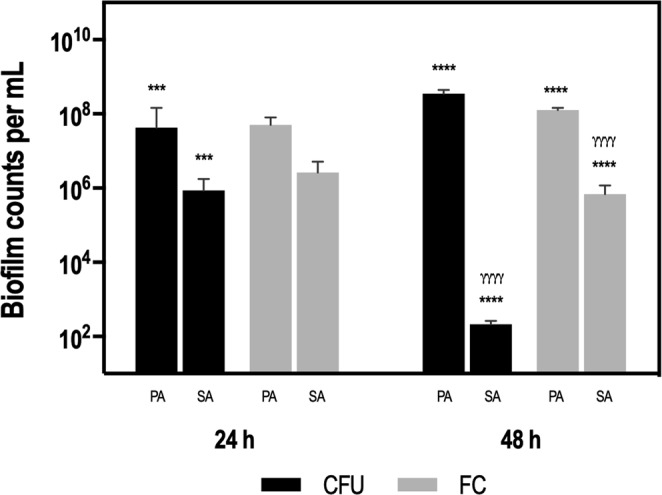
Quantification of *P*. *aeruginosa* and *S*. *aureus* populations in dual-species biofilms by plate count (CFU) and flow-cytometry (FC), following 24- and 48-h of growth. Biofilm counts are expressed as means ± SD of CFU per mL or cells per mL, respectively. For each condition three independent experiments were performed. Statistical significance was determined by performing an ANOVA followed by a Tukey’s multiple comparison test to compare: (*) significantly different *P*. *aeruginosa* counts *versus S*. *aureus* counts for each method; ***P < 0.001; ****P < 0.0001. (^γ^) significantly different *P*. *aeruginosa* counts between methods and significantly different *S*. *aureus* counts between methods; ^γγγγ^P < 0.0001. Abbreviations: PA = *P*. *aeruginosa*, SA = *S*. *aureus*.

Dual-species biofilms were first characterized over the course of 24- and 48-h by enumerating *P*. *aeruginosa* and *S*. *aureus* through CFU assessment on selective media (PIA and MSA, respectively) and also by using flow-cytometry (Fig. 1). At both time points, *P*. *aeruginosa* was the prevalent species detected, being the differences between *P*. *aeruginosa* and *S*. *aureus* more relevant at 48-h of growth. Interestingly, these differences were more dramatic (~6 log_10_ CFU/mL) when considering culture quantification only, but significantly lower (~2 log_10_ cells/mL) when quantified by flow- cytometry. These results highlight that culture methods are not reliable to analyse biofilm population composition in certain conditions^11^. This has been justified by the fact that, with time, bacteria within biofilms can enter a VBNC state and, therefore, are not detectable by conventional CFU plating^35^.

In order to analyze and compare the biofilm structures, 24- and 48-h dual-species biofilms were directly examined using scanning electron microscopy (Supplementary Figure S1). The dual-species consortium showed, at 24-h, a non-contiguous layer of cells, that evolved, at 48-h, into mature biofilm producing a thick layer of co-aggregated cells surrounded by extracellular matrix. Both *P*. *aeruginosa* (road-shape) and *S*. *aureus* (coccus-shape) were distinguishable at 24-h but at 48-h, the dense structure of the biofilm did now allow to distinguish the bacterial species.

### The impact of RNA extraction variability in the utilization of 16S rRNA as a direct quantification tool

Molecular-based methodologies have greatly increased our understanding regarding ecological interactions occurring among members of polymicrobial communities, when compared to culture-based methods^36^. However, those techniques are not void of limitations and, especially when using RNA-based approaches, several controls and normalizations need to be considered^37–41^.

**Figure 2:**
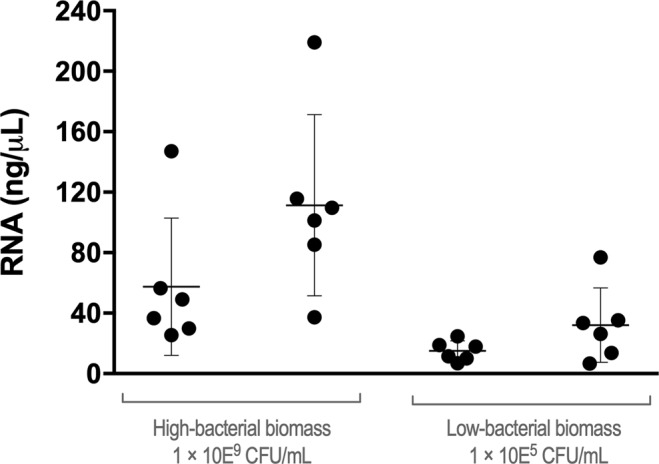
Total RNA extraction variability obtained from 10^9^ CFU/mL and 10^5^ CFU/mL of *P*. *aeruginosa* planktonic cultures. For both high- and low-bacterial concentrations, two independent biological samples were experienced, each one aliquoted in six technical replicates.

A key aspect in RNA-based qPCR quantification is the need to normalize RNA extraction efficiencies, since the quality and quantity of extracted RNA can affect downstream applications. While often underreported, RNA extraction efficiency varies^38,39^. To demonstrate this, we first assessed the variability of RNA extraction processes, followed an optimized protocol previously described^39,42^. Briefly, we split planktonic suspensions of either high or low cell concentrations of *P*. *aeruginosa*, in six aliquots from the same biological sample, to perform total RNA extraction. As observed in Fig. 2, a high variability of total RNA was obtained, especially when using high density populations. Total RNA concentrations ranged from 26 to 220 ng/μL in the 10^9^ CFU/mL aliquots and 7 to 77 ng/μL in the 10^5^ CFU/mL aliquots. Noteworthy, aliquots of the same biological sample yielded distinct RNA concentrations, with a higher inter-assay variability in high biomass samples (coefficient of variation of 0.66) compared to the low biomass (0.23) (Supplementary Table S1).

**Figure 3:**
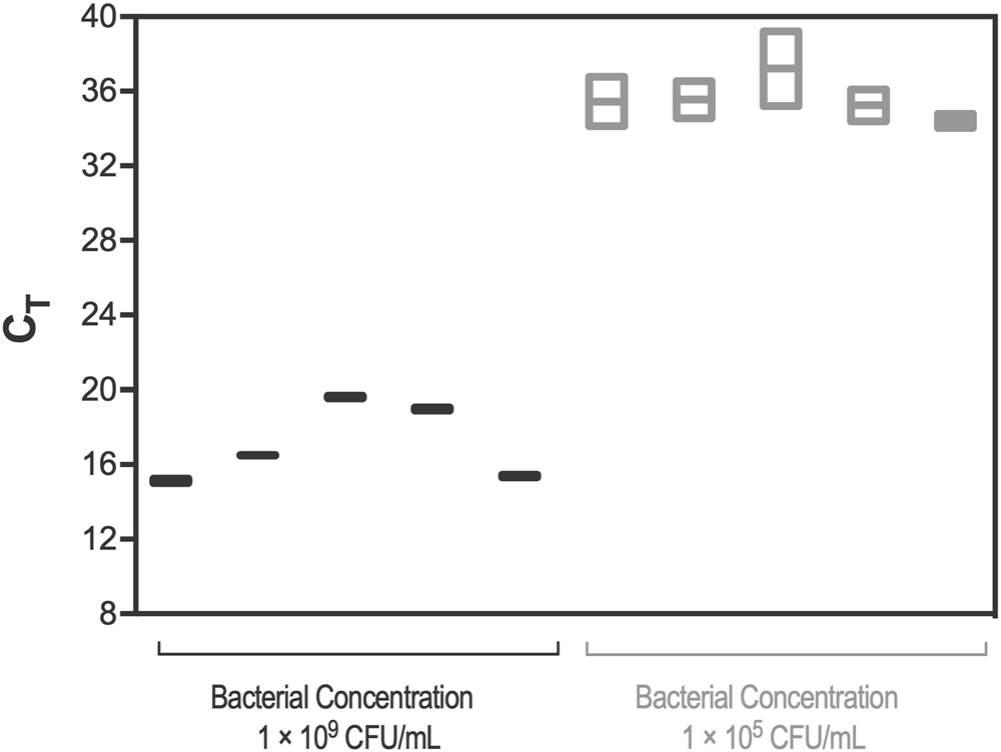
Variability in cycle threshold (Ct) values for *16S rRNA* gene from 10^9^ CFU/mL and 10^5^ CFU/mL of *P*. *aeruginosa* planktonic samples corresponding to high- and low-bacterial concentrations. For each bacterial concentration five technical replicates from the same biological sample were used.

Next, we quantified *16S rRNA* for each aliquot. Not surprising, the cycle threshold (C_t_ value) detection was highly variable, in a range of 3 to 5 cycles (Fig. 3). These results confirmed that quantifying *16S rRNA*-target, by itself, is not a good strategy to quantify bacterial communities.

### Impact of utilizing an external *ref* mRNA in bacterial quantification using 16 sRNA

When quantifying gene expression by qPCR, a normalization strategy is required and often it includes the quantification of a reference gene, also known as housekeeping gene^43,44^. Due to the variability of the experimental method^39^ the same principle needs to be applied when quantifying bacterial populations by qPCR. The most common alternative is to normalize the levels of the target mRNA to that of an exogenous mRNA spike-in transcript^45–53^. The exogenous mRNA control is characterized by: (i) not being originally found in the sample, (ii) its addition should be previous to the RNA extraction procedure and (iii) its initial concentration is known^54^. After laboratorial sample processing, the amount of recovered exogenous mRNA is determined by qPCR. Although exogenous *ref m*RNA approach has been previously used^45–53^, to the best of our knowledge, this study is the first evaluating polymicrobial biofilm samples.

**Figure 4:**
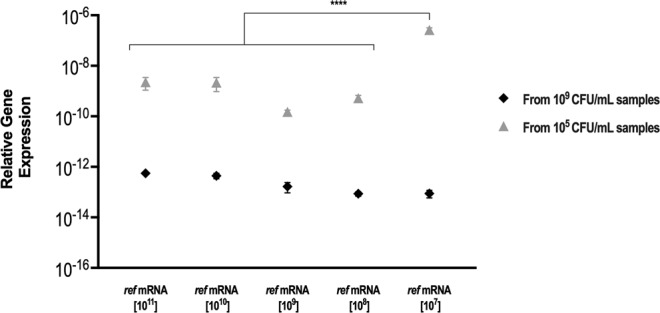
Effect of the *ref* mRNA normalization on the *16S rRNA* expression levels. Relative gene expression of the *16S rRNA* normalized to the *ref* mRNA, using the delta Ct method, in 10^9^ CFU/mL and 10^5^ CFU/mL samples adjusted to the initial copies of *ref* mRNA used. For each bacterial concentration five technical replicates were spiked with standard concentrations of *ref* mRNA prior to RNA extraction and subsequent reverse transcription. For each condition two independent biological samples were used. Statistical significance within each samples’ set was determined by performing one-way ANOVA analyses followed by a Tukey’s multiple comparison test (****P < 0.0001).

In this study, commercial luciferase mRNA (*ref* mRNA) was preferred as the exogenous reference gene since the specific sequence is not found in any of the bacterial species tested, as assessed via NCBI GenBank database (www.ncbi.nlm.nih.gov). To quantify the *ref* mRNA, a standard curve was constructed, by plotting the Ct values against the known initial *ref* mRNA copy number (Supplementary Figure S2A). The standard curve generated covers a linear range along the selected five orders of magnitude with a correlation coefficient (R^2^) of 0.977, suggesting that all of the tested concentrations can be used in the subsequent analysis. Nevertheless, considering that the initial concentration of *ref* mRNA spiked into the samples may interfere with the mRNA quantification due to competition for qPCR reagents^51^, five aliquots derived from the same *P*. *aeruginosa* biological culture were amended with 1 × 10^7^ to 1 × 10^11^ copies of *ref* mRNA, immediately after cell lysis (Supplementary Figure S2B). The significance of correlation between the total copies of *ref* mRNA added and total copies of *ref* mRNA extracted from each sample was calculated based on Pearson’s coefficient (r = 0.99, P < 0.05 for 10^9^ CFU/mL samples and r = 0.90, P < 0.05 for 10^5^ CFU/mL samples). Next, in order to test the sensitivity and accuracy of the qPCR assay, the *16S* *rRNA* gene was normalized to the exogenous control. This was done either in the 10^9^ and 10^5^ CFU/mL samples. Figure 4 demonstrates that in almost all conditions tested, no significant variations were found in the relative gene expression determined. The exception was in the low biomass sample amended with 1 × 10^7^ copies of *ref* mRNA. This suggests that at low amounts of genomic material, the efficiency drops below the linear range of the method^55^.

For further analysis, we selected the *ref* mRNA concentration of 1 × 10^8^ copies of *ref* mRNA/μL, since under these conditions the best mRNA recovery was found, for either the low or high cell concentrations (Supplementary Table S2). Interestingly, the low mRNA recovery estimated confirms that somewhat random physical losses during RNA isolation will impair some quantification approaches^24,37^. Overall, these results support the importance of a *ref* mRNA normalization for proper quantification of bacterial populations using the RNA-based method.

### Validation of the *ref* mRNA normalization in controlled dual-species communities

**Figure 5:**
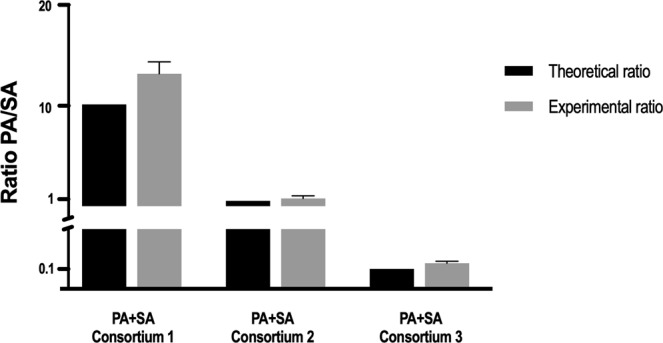
Comparison between the theoretical and the experimental qPCR PA/SA ratio of dual-species planktonic consortia (PA/SA ratios of 10, 1 and 0.1). For each condition two independent experiments were performed, each one with two technical replicates. Statistical significance was determined by performing independent ANOVA analyses followed by a Dunnett’s multiple comparison test.

To assess the robustness of this approach, the relative expression of the *16S rRNA* gene was analysed in three well defined dual-species planktonic samples, (*P*. *aeruginosa/S*. *aureus* ratio equal to 10, 1 and 0.1). The C_T_ of each *16S rRNA* target was normalized to the *ref* mRNA in order to determine the experimental ratios, calculated by applying the equation described in the section *Quantitative Real Time-PCR* of the *Experimental procedures* (Supplementary Table S3). As shown in Fig. 5, the experimental ratios obtained by applying the *ref* mRNA strategy were consistent with the theoretical ratios for all *P*. *aeruginosa* and *S*. *aureus* populations. Statistical analysis showed no significant differences between the theoretical and the experimental PA/SA ratios (P > 0.05).

### Quantification of a dual-species biofilm communities by RNA-based qPCR

**Figure 6:**
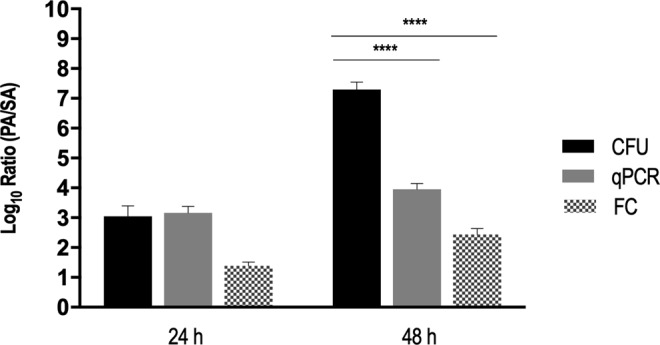
Relative quantification of 24-h and 48-h-old dual-species biofilms by plate count, qPCR and flow-cytometry. For each condition three independent experiments were performed. Statistical significance was determined by performing independent ANOVA analyses followed by a Dunnett’s multiple comparison test (****P < 0.0001).

Following the experimental validation of the *ref* mRNA normalization strategy in the well-defined planktonic cultures, 24- and 48-h-old dual-species biofilms of *P*. *aeruginosa* and *S*. *aureus* were formed and quantified by qPCR (Supplementary Table S4). The same biofilms were also characterized by culture dependent (plate count) and culture independent (flow cytometry) methods (Supplementary Table S5). As shown in Fig. 6, the ratios of *P*. *aeruginosa*/*S*. *aureus* varied significantly accordingly with the method used, especially in the 48-h-old biofilm (P < 0.0001). The differences between culture-dependent and culture-independent methods were more significative than between both culture-independent methods. Interestingly, both qPCR and flow-cytometry methods have ascertained *P*. *aeruginosa* as the predominant species in the biofilm, but a significant population of *S*. *aureus* was also detected by these two methods in the 48-h-old consortium, in striking contrast with the CFU quantification. It is conceivable that after 48-h of co-culture with *P*. *aeruginosa*, a remarkable portion of *S*. *aureus* cells acquire a VBNC state. Indeed, as biofilms mature, often portions of bacteria within the community enter a VBNC state, in response to stressful conditions, such as starvation^56^ or antimicrobial treatments^8,57,58^, suggesting that this is an adaptive strategy for long-term survival under unfavourable conditions^59^. Although a series of physiological changes occur during the transition from cultivable to VBNC state, VBNC cells were found to maintain high levels of rRNA, similar to cultivable cells^19,60^, being qPCR RNA-based assays been commonly used to evaluate gene expression profile in cultures containing VBNC cells^61–63^. However, an important technical limitation of these studies is that transcription is evaluated as the global gene expression within the biofilm, not able to differentiate the physiological state of sub-populations. To determine the differences within such populations, the new generation of single-cells transcriptomic analysis^64^ should be used, but as far as we are aware, this has not been attempted in dual-species biofilms.

We also performed a Live/Dead (L/D) viability assay on disrupted 48-h-old dual-species consortia, to inspect the overall biofilm-cell viability (Supplementary Figure S3). L/D staining showed that cocci-like bacterial cells were more abundant in the green than the red filter. Those results reinforce the previous conclusion that even though *S*. *aureus* lost its culture capability after 48-h of co-culture with *P*. *aeruginosa*, some bacteria remains in a viable state.

### Applicability of the RNA- based qPCR quantification method to further study inter-species microbial interactions

A potential advantage of the RNA-based qPCR quantification approach to study dual-species biofilms, is its ability to not only discern between the relative proportions of each species, but also to determine if a specific gene is being induced or repressed. To achieve this, the gene of interest should be normalized to the reference gene (in this case, *16s RNA* gene), while this reference gene is also normalized against the exogenous *ref* mRNA (in this case luciferase) in order to account for the relative quantification of each bacterial population.

**Figure 7:**
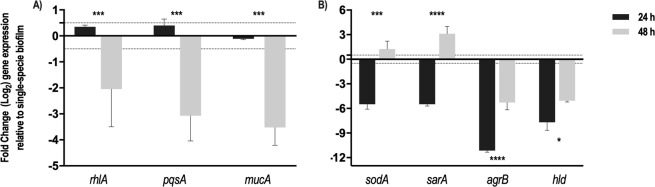
Gene expression profile of (**A**) *P*. *aeruginosa* and (**B**) *S*. *aureus* virulence-related genes in 24- and 48-h-old dual-species biofilms, as compared to single-species biofilms. Transcripts were normalized to *16S rRNA* and results were calculated as fold change relative to gene expression values obtained for single-species biofilms. Dashed lines represent a 2-fold cut-off value. For each condition at least two independent experiments were performed with three technical replicates. Statistical significance was determined by performing an unpaired two-tailed *t* test (***P < 0.001).

As a demonstration of principle of this method potential, we quantified the differences in expression of key virulence genes of *P*. *aeruginosa* and *S*. *aureus*, between single- or dual-species biofilms. To inspect the impact of *S*. *aureus* on *P*. *aeruginosa* virulence potential, we analysed the expression of *pqsA* (Pseudomonas quinolone signal), *rhlA* (rhamnolipids) and *mucA* (alginate biosynthesis). Pseudomonas quinolone signal (PQS) is an important *P*. *aeruginosa* signalling molecule regulating the production of quorum-sensing (QS) dependent virulence factors, such as elastase, pyocyanin and rhamnolipids^65^. Rhamnolipids are biosurfactants produced by *P*. *aeruginosa* and several bacterial species acting as immune modulators and virulence factors; have antimicrobial activities and are involved in surface motility and in bacterial biofilm development^66^. Alginate is an important extracellular virulence factor and has been shown to impair host innate defences related to phagocytes^67^. Chronic *P*. *aeruginosa* infections are often associated with a mucoid phenotype due to the production of large quantities of the exopolysaccharide alginate^68^. The switch from nonmucoid to mucoid state is induced by the inactivation of *mucA* gene^69^. Notably, our results indicated that in 48-h-old dual-species biofilms, the expression levels of *P*. *aeruginosa pqsA*, *rhlA* and *mucA* were down-regulated, as compared with *P*. *aeruginosa* single-species (Fig. 7A). Interestingly, these differences were not detected in early (24-h-old) biofilms, suggesting a biofilm maturation-dependent effect.

Additionally, the role of *P*. *aeruginosa* in the gene expression profile of select *S*. *aureus* QS- (*agrB* and s*arA*) and virulence-related genes (*sodA* and *hld*) was also assessed. Agr and Sar are two main QS systems in *S*. *aureus* that control the expression of several virulence genes and production of cell-wall-associated proteins^70,71^. The *hld* gene, codifying delta hemolysin, is positively controlled by *agr* system and plays a role in severe staphylococcal infections^72^. Superoxide dismutase is a metalloprotein, encoded by the *sodA* gene, that inactivates harmful superoxide radicals encountered during oxidative stress^73^. Under the tested conditions, the presence of *P*. *aeruginosa* promoted the downregulation of *sodA*, *sarA*, *agrB* and *hld* genes in 24-h dual-species biofilms, while for 48-h-old dual-species biofilms *sodA* and *sarA*, were up-regulated (Fig. 7B).

### Major conclusions and study limitations

Our results underline the importance of selecting appropriate methodologies to analyse dual-species biofilm communities, since some techniques, if used alone, may not provide accurate insights into biofilm composition, resulting in misleading conclusions. Both culture-independent techniques used in this study provided a more rapid and sensitive assessment of the individual species present in the dual-species biofilm consortia, comparing with conventional culture, which underestimated the real abundance of *S*. *aureus* population. As each technique has its particularity, Table 1 summarise the advantages, disadvantages, and purposes of use of all methodologies applied in this study.

**Table 1:** Comparison of the methodologies described in this study, conventional culture, flow-cytometry and qPCR. Advantages, disadvantages and purposes of use applicable to microbial communities’ analysis.

	Conventional Culture	Flow Cytometry	qPCR
Advantages	♦ Standardized method that allows the identification of the most abundant members^78,79^.	♦ Allows a rapid detection of multi-species samples^80–82^.	♦ Detection of live cells^18,84^.
		♦ High specificity and sensitivity to detect both viable and VBNC cells^83^.	♦ Can be used for different applications: bacterial detection^85^ and to assess changes in the gene expression profile^86^.
Disadvantages	♦ Time consuming analysis;	♦ Require full optimization of the method to each species^81^.	♦ optimization (primers specificity, RNA extraction)^42,43,87^.
	♦ Requires selective media^87^;		
	♦ Not detection of cells in a viable but non-cultivable state^7,35^.	♦ Limit of cell detection^81^.	
Purposes of use	Screening method for bacterial enumeration.	Fast method for enumeration of a large number of samples.	Fast method for detection and quantification;
			Can simultaneously analyse gene expression profile of key genes (e.g. virulence genes) in the tested samples.

Of relevance, our experiments demonstrated the advantage of using a RNA-based qPCR quantification and highlighted how important is the use of an appropriate mRNA control molecule in the qPCR assay, in order to address experimental biases. Overall, this experiment confirmed that this qPCR method is able to assess specific gene expression profiling together with quantification of bacterial populations in dual-species biofilms. Despite the advantages of the qPCR assay optimized through this study, it must be recognized that potential bias can also be introduced. For instance, when gene transcript abundance is calculated by normalization against the *ref* mRNA, the assumption is that it reflects the behaviour among the whole RNA pool over the entire sample’s processing steps. One potential bias in normalization against the *ref* mRNA is that it cannot compensate the differences of RNA extraction and reverse transcription efficiencies between the targeted species. While the occurrence of this bias cannot be excluded under the tested conditions, it is believed that is safe to assume that it is not very significant, given the results achieved. The qPCR sensitivity to quantify species with very low cell numbers^74^ in certain scenarios (e.g. after exposure of an antimicrobial therapy targeting specifically a member of the polymicrobial community) may also limit its usefulness on the quantification of less prevalent species within the community.

## Experimental Procedures

### Bacterial strains and culture conditions

*P*. *aeruginosa* (strain UCBPP-PA14) and *S*. *aureus* (strain ATCC 25923) were used throughout this work. Both strains were stored at −80 ± 2 °C in Tryptic Soy Broth (TSB, Liofilchem) supplemented with 20% glycerol. Prior to each assay, bacteria were subcultured from frozen stock preparations onto plates of TSB supplemented with 2% (W/V) agar (TSA) and incubated aerobically at 37 °C for 24-h.

### Planktonic growth

**Figure 8:**
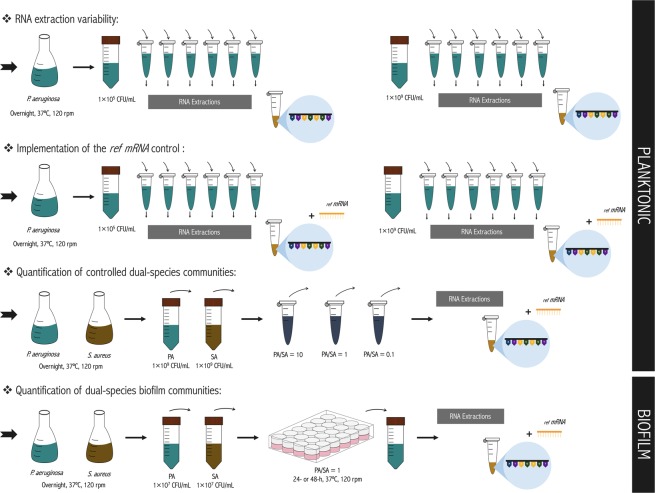
Experimental workflow of the planktonic and biofilm samples preparation.

All assays were carried out by using a standardized bacterial inoculum. Briefly, a few colonies of each species were collected from the TSA plates and grown overnight in batches of TSB (Liofilchem) at 37 °C under agitation (120 rpm). Subsequently, cells were harvested by centrifugation (9000 *g*, 5 min) and washed in sterile saline solution (0.9% NaCl). The concentration of cellular suspensions was then adjusted in TSB by spectrophotometric measurement at 640 nm (calibrations were performed for each bacterial strain to relate the absorbance at 640 nm with the number of colony forming units, CFU) to obtain a final concentration of 1 × 10^5^ CFU/mL or 1 × 10^9^ CFU/mL. Unless otherwise stated, two independent assays with five to six replicates for each condition tested were performed. For the preparation of the dual-species planktonic suspension, overnight grown cultures of each species were adjusted to a final concentration of 1 × 10^9^ CFU/mL and then mixed in three samples of various compositions (*P*. *aeruginosa*/*S*. *aureus* ratios of 10, 1 and 0.1). Two independent assays for each condition with two replicates were used. To perform the RNA extraction and subsequent reverse transcription, single- and dual-species planktonic suspension were centrifuged (7000 rpm, 10 min, 4 °C) and the pellet resuspended in RNA protect (Qiagen), as described below. Planktonic samples preparation is schematically represented in Fig. 8.

### Biofilm formation

Dual-species biofilms encompassing *P*. *aeruginosa* and *S*. *aureus* were grown as previously described, with minor modifications^4^. Briefly, overnight cultures of each strain, grown in TSB at 37 °C and 120 rpm in aerobic conditions, were washed in sterile water and diluted in TSB to obtain 1 × 10^7^ CFU/mL as final concentration. The dual-species cultures were then prepared by mixing the inoculum of each species in a 1:1 ratio. Each well of a 24-well polystyrene plate (Orange Scientific, Braine-l’Alleud, Belgium) was seeded with 1 mL of *P*. *aeruginosa* and *S*. *aureus* suspension and incubated at 37 °C on a horizontal shaker (120 rpm) for 24- and 48-h. Following biofilm growth, the planktonic fraction of the polystyrene plates was removed and the wells were washed twice with distilled sterile water. Biofilms cells were resuspended in 1 mL of 0.9% NaCl, detached by scraping and collected by centrifugation (7000 rpm, 10 min, 4 °C). Three biologic replicates of each condition were performed. To perform the RNA extraction and subsequent reverse transcription, a biofilm pooling (10 wells of a 24-well-plate) was resuspended in RNA protect (Qiagen). Biofilm samples preparation is schematically illustrated in Fig. 8.

### RNA extraction

RNA was extracted using the RNeasy mini kit (Qiagen), as previously optimized^42^. Briefly, bacteria were first resuspended in 500 μL lysis buffer (provided in the kit). Then, 500 μL of phenol solution (AppliChem) was added, and the entire suspension was transferred to a safe lock tube (2 mL) with 0.4 g of acid-washed 150–212 mm silica beads (Sigma). The tube content was vortexed for 20 s previously to being placed in the FastPrepH cell disruptor (BIO 101, Thermo Electron Corporation, Thermo Scientific) at 6.5 m/s for 35 s. The samples were then cooled on ice and the beat- beading step repeated twice. Afterwards, Afterwards, 700 μL of 70% ethanol (Fisher Scientific) was added to the supernatants, followed by 1 μL of 1 × 10^8^ luciferase control RNA transcripts (*ref* mRNA) (Promega). To achieve the final RNA fraction, 50 μL of RNase-free water was added to the RNA columns. To digest possible contaminating genomic DNA, RNA was treated with DNase I (Fermentas, Ontario, Canada) following manufacturer instructions. Briefly, the RNA samples were incubated at 37 °C for 30 min after the addition of 2 μL of DNase I and 5 μL of reaction buffer. Then, 5 μL of 25 mM EDTA was added to the mixture and incubated at 65 °C for 10 min to inactivate the DNase I enzyme.

### RNA quality determination

RNA integrity and purity were assessed by gel electrophoresis and the total RNA concentration was spectrophotometrically determined using a NanoDrop 1000^TM^ (Thermo Scientific). As indicator of protein contamination and polysaccharide, phenol, and/or chaotropic salt contamination, the absorbance ratio A_260_/A_280_ and A_260_/A_230_ were measured, respectively^75^. The RNA integrity was assessed by 23S/16S banding pattern visualization. Electrophoresis was carried out at 80 V for 60 min using a 1.5% agarose gel. The gel was stained with GelRed (Thermo Fisher Scientific) and visualized using a GelDoc2000 (Bio-Rad, Hercules, CA, US) (Supplementary Figure S4). RNA was stored at −80 °C for further use.

### Determination of RNA recovery using an exogenous internal reference mRNA

Luciferase mRNA (Promega) was used as the exogenous internal reference (*ref* mRNA) to estimate the percentage of RNA loss during extraction. After the centrifugation of the lysed cells, 1 μL of a range of 1 × 10^11^ to 1 × 10^7^ *ref* mRNA transcripts/μL were added to the aqueous phase. After RNA extraction, the amount of *ref* mRNA recovered from samples was measured by qPCR and the standard curve generated from serial dilutions of *ref* mRNA. The recovery fraction was calculated by dividing the determined levels by the initial number of *ref* mRNA transcripts added to the samples.

### Quantitative Real Time-PCR (qPCR)

For the quantification of the *16S* *rRNA* by qPCR, total RNA was reversely transcribed to complementary DNA (cDNA) using GRS cDNA Synthesis Kit (Grisp). Specifically, 100 ng of total RNA was reversely transcribed in 10 μL of reaction volume by using random primers. The samples were incubated at 65 °C for 5 min, 37 °C for 60 min, and 70 °C for 10 min. Primers, specific for *16S rRNA*, housekeeping gene, and virulence-related genes of *P*. *aeruginosa* and *S. aureus* were designed (Supplementary Table S5). The qPCR reaction was performed by mixing together 5 µL of master mix SYBR Xpert Fast SYBR (Grisp), 2 µL of 1:100 diluted cDNA, 0.5 µL of forward and reverse primes (5 µM), and water up to a total volume of 10 µL. No reverse transcriptase (NRT) and a no template control (NTC) were included to verify the reaction mixtures were DNA and other contaminants-free. The efficiency of the primers used was measured by the dilution method^38^. To perform the qPCR run, a CFX 96 (Bio-Rad) was used with the following cycle parameter: 95 °C for 3 minutes, 40 cycles of 95 °C for 5 s, and 60 °C for 20 s. qPCR products were analysed by melting curves for unspecific products or primer dimer formation. The qPCR was applied to independently quantify the 16S *rRNA* of *P*. *aeruginosa* and *S*. *aureus* and the *ref* mRNA in each cocktailed mixed sample. The normalized gene expression was determined by using the delta Ct method (2^∆Ct^), a variation of the Livak method, where ∆Ct = Ct (*ref* mRNA) − Ct (16S *rRNA*). After normalization of the 16S *rRNA* for both species in each cocktail sample, the PA/SA ratio was calculated by applying the following equation:

$$\begin{array}{c}{Ratio}(\frac{{PA}}{{SA}})=\frac{{Normalized}\,[{\rm{ref}}\,{\rm{mRNA}}/{16S}\,{rRNA}\,(\text{PA})]}{{Normalized}\,[{\rm{ref}}\,{\rm{mRNA}}/{16S}\,{rRNA}\,(\text{SA})]}\\ \,\times \,{Normalized}\,[{16S}\,{rRNA}\,(\text{PA})/{16S}\,{rRNA}\,(\text{SA})]\end{array}$$

Relative fold increase of *pqsA*, *rhlA*, *mucA*, *sodA*, *sarA*, *agrB* and *hld* genes, was determined by applying the Pfaffl equation^76^ using *16S rRNA* as reference gene. At least two biologic replicates of each condition were used with three technical replicates.

### Biofilm cultivability

The number of adhering bacteria was determined after biofilm cell detachment. To remove any aggregates, biofilm suspensions were first sonicated for 10 s at 30% amplitude^11^ (Cole Parmer Ultrasonic Processor, IL, USA) and then the culturable cell count was carried out. Briefly, the disrupted biofilms were serially diluted (1:10) in 0.9% NaCl, streaked onto different selective agar media and incubated at 37 °C for 18–24 h, for CFU counting. Pseudomonas isolation agar (PIA) and Mannitol Salt Agar (MSA) were the selective media used to discriminate *P*. *aeruginosa* and *S*. *aureus*, respectively. Values of cultivable biofilm cells were expressed as CFU/mL and represented as the average of three independent experiments.

### Flow-cytometry

The total number of *P*. *aeruginosa* and *S*. *aureus* cells in 24- and 48 h-old mixed biofilms was determined using flow-cytometry. In brief, biofilm cells were collected by centrifugation (7000 rpm, 10 min, 4 °C) and suspended in 100 µL of a solution with 25 µg/mL of wheat germ agglutinin (WGA) conjugated with FITC (Molecular probes, ThermoFisher Scientific). This suspension was incubated at room temperature, in the dark, for 15 min. Thereafter, cells were washed twice with 0.9% NaCl, suspended in 1 mL of the same solution and sonicated for 10 s at 30% amplitude (Cole Parmer Ultrasonic Processor, IL, USA) to disrupt aggregates. The bacterial fluorescence analysis was carried out using an EC800 Sony flow cytometer (Sony Biotechnologies Inc., CA, USA) equipped with a 488 nm laser. Multiparametric analyses were performed on both scattering signals (FSC, SSC) and FL1 channel. All the detectors were set to logarithmic amplification. Samples were acquired with a flow rate of 10 μL/min and the analysis stopped when 80 000 events were detected. Three biologic replicates of each condition were used and each sample was analysed at least twice to ensure an accurate counting. Data analysis was performed using EC800 analysis software (Sony Biotechnologies).

### Assessment of cell viability by Live/Dead staining

To analyse the viability of dual-species biofilm consortia 48-h-old biofilms were scraped, and the biofilm disrupted-cells were collected in 1 mL sterile distilled water. Afterwards, disrupted biofilm cells were stained in the dark, for 15 min, with SYTO® BC (2 μM) and Propidium Iodide (15 μM) (both from Invitrogen™, CA, USA) prepared in saline solution, in order to differentially label live and dead cells by microscopy. For microscopic observation, an Olympus BX51 microscope fitted with fluorescence illumination was used. The optical filter combination consisted of 470 to 490 nm in combination with 530 to 550 nm excitation filters.

### Scanning electron microscopy

Scanning electron microscopy (SEM) was used to examine the dual-species biofilm as described before, with minor modifications^77^. Briefly, dual-species biofilms were formed on Thermanox® plastic coverslips (Thermo Scientific™; Rochester, NY, USA) placed in the bottom of the wells of 24-well microtiter plates (Orange Scientific, Belgium). Prior to SEM observation, discs were added to aluminum pin stubs with electrically conductive carbon adhesive tape (PELCO Tabs™). Samples were coated with 2 nm of Au for improved conductivity. The aluminum pin stub was then placed inside a Phenom Standard Sample Holder or Phenom Charge Reduction Sample Holder. The analysis was conducted at 5 kV with intensity image. The samples were characterized using a desktop Scanning Electron Microscope (SEM) (Phenom ProX, Netherlands). All results were acquired using the ProSuite software.

### Statistical analysis

All assays were carried out in triplicate and repeated at least twice, and the results are presented as means ± SDs. The Pearson’s correlation coefficient was calculated to determine the association between two variables. All P-values were based on two-tailed tests of significance, and confidence interval of 95% was used in all the analysis performed. Statistical analysis was performed using Graph Pad Prism 7.0.

## Supplementary information

Supplementary material

## Data Availability

The datasets generated during and/or analysed during the current study are available from the corresponding author on reasonable request.
