# Supplementary material for: RNA-based qPCR as a tool to quantify and to characterize dual-species biofilms
Source: Sci Rep. 2019 Sep 20;9:13639. doi: 10.1038/s41598-019-50094-3 (PMC6754382; doi:10.1038/s41598-019-50094-3)
Supplement: Supplementary file 1 — Supplementary material [file 41598_2019_50094_MOESM1_ESM.docx]

Supplementary Information

**RNA-based qPCR as a tool to quantify and to characterize dual-species biofilms**

Andreia Patrícia Magalhães^1^, Ângela França^1^, Maria Olívia Pereira^1^ and Nuno Cerca^1#^

^1^Centre of Biological Engineering, LIBRO – Laboratório de Investigação em Biofilmes Rosário Oliveira, University of Minho, Campus de Gualtar, 4710-057 Braga, Portugal

**^#^Corresponding author details**: Nuno Cerca. Centro de Engenharia Biológica, Universidade do Minho, Campus de Gualtar, 4710-057 Braga, Portugal. Tel: +351-253-60443; Fax: +351-253-678-986; E-mail: [nunocerca@ceb.uminho.pt](mailto:nunocerca@ceb.uminho.pt)

# Supplementary Figures

**Supplementary Figure S1.** Scanning electron microscopy of 24- and 48-h-old dual-species biofilms of *P. aeruginosa* (PA) and *S. aureus* (SA). ﻿The bar corresponds to 30 μm (magnification of 2750x).

**Supplementary Figure S2.** **(A)** **Titration curve showing the decrease in cycle threshold (C_t)_ for the exogenous reference mRNA (*ref* mRNA) as the number of *ref* mRNA transcripts reversely transcribed increases.** The *ref* mRNA was first reversely transcribed to complementary cDNA and then amplified by qPCR. Data points of two independent dilution series. **(B)** **Recovery of *ref* mRNA after sample spiking with standard concentrations.** Five planktonic samples were spiked with standard concentrations of *ref mRNA* prior to RNA extraction and reverse transcription. Linear regression between total copies of *ref* mRNA added and total copies of *ref* mRNA extracted from **(B1)** high-bacterial biomass samples (R^2^=0.97, slope 0.77, intercept 0.80) and **(B2)** low-bacterial biomass samples (R^2^=0.82, slope 0.52, intercept 2.23). 95% confidence interval for fitted line is presented with grey area.


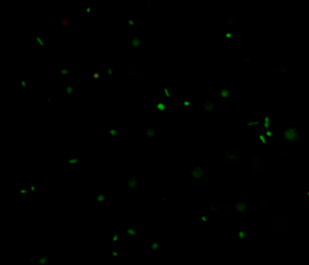

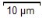

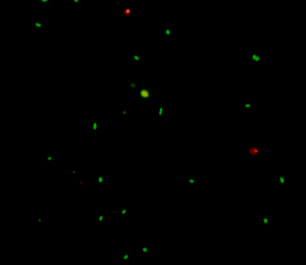

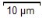

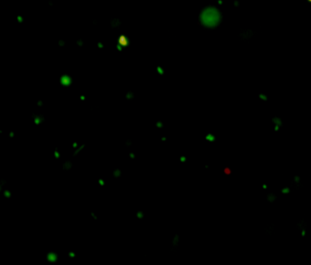

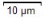

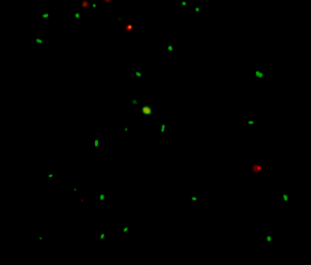

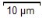


**Supplementary Figure S3 -** Examples of fluorescence microscopy images used to evaluate the bacterial viability of disrupted 48-h-old dual-species biofilm consortium, ﻿stained with LIVE/DEAD staining system. ﻿Dead bacteria fluoresce red and viable bacteria fluoresce green.

**
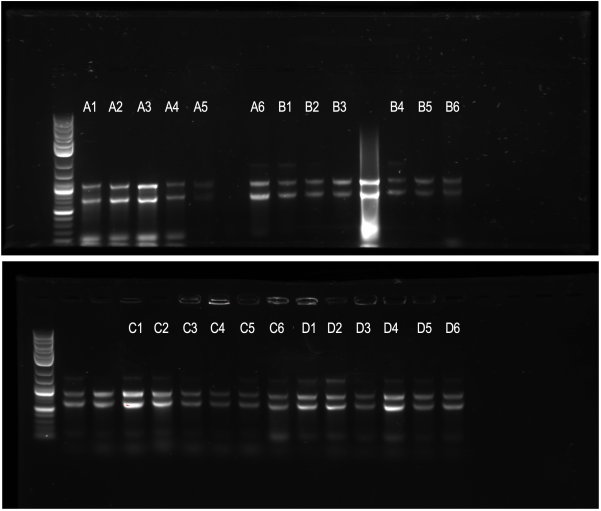
**

**Supplementary Figure S4** - RNA integrity determined by visualization in GelRed stained agarose gel. **A1-A6** and **B1-B6:** *P. aeruginosa* samples with 1×10^9^ CFU/L. **C1-C6** and **D1-D6:** *P. aeruginosa* samples with 1×10^5^ CFU/L.

# Supplementary Tables

**Supplementary Table S1** - RNA concentration and quality of *P. aeruginosa* planktonic samples. For each condition (1×10^9^ CFU/mL and 1×10^5^ CFU/mL) two independent biological samples were aliquot in six technical replicates, before RNA was extracted.

| **Concentration** | **Sample** | | **RNA (ng/μL)** | | **A260/280** | **A260/230** | | **Concentration** | | **Sample** | | | | **RNA (ng/μL)** | | **A260/280** | | **A260/230** | |  |
| --- | --- | --- | --- | --- | --- | --- | --- | --- | --- | --- | --- | --- | --- | --- | --- | --- | --- | --- | --- | --- |
| 1×10^9^ CFU/mL | BiologicalSample 1 | Replicate 1 | | 49.05 | 1.78 | 0.44 | | 1×10^5^ CFU/mL | | Biological Sample 1 | | Replicate 1 | | 24.75 | | | 1.55 | 0.12 | |  |
|  |  | Replicate 2 | | 29.85 | 1.73 | 0.21 | |  |  |  |  | Replicate 2 | | 11.40 | | | 1.67 | 0.10 | |  |
|  |  | Replicate 3 | | 56.50 | 1.68 | 0.25 | |  |  |  |  | Replicate 3 | | 6.85 | | | 1.48 | 0.10 | |  |
|  |  | Replicate 4 | | 25.55 | 1.55 | 0.60 | |  |  |  |  | Replicate 4 | | 18.95 | | | 1.63 | 0.10 | |  |
|  |  | Replicate 5 | | 36.65 | 1.72 | 0.77 | |  |  |  |  | Replicate 5 | | 10.20 | | | 1.53 | 0.20 | |  |
|  |  | Replicate 6 | | 147.10 | 1.72 | 0.51 | |  |  |  |  | Replicate 6 | | 17.90 | | | 1.46 | 0.15 | |  |
| 1×10^9^ CFU/mL | Biological Sample 2 | Replicate 1 | | 37.30 | 1.70 | 0.35 | | 1×10^5^ CFU/mL | | Biological Sample 2 | | Replicate 1 | | 6.70 | | | 1.48 | 0.10 | |  |
|  |  | Replicate 2 | | 109.65 | 1.81 | 0.88 | |  |  |  |  | Replicate 2 | | 76.95 | | | 1.43 | 0.39 | |  |
|  |  | Replicate 3 | | 219.15 | 1.70 | 0.55 | |  |  |  |  | Replicate 3 | | 13.65 | | | 1.37 | 0.17 | |  |
|  |  | Replicate 4 | | 85.25 | 1.80 | 0.58 | |  |  |  |  | Replicate 4 | | 33.45 | | | 1.35 | 0.39 | |  |
|  |  | Replicate 5 | | 115.65 | 1.86 | 1.16 | |  |  |  |  | Replicate 5 | | 35.40 | | | 1.38 | 0.40 | |  |
|  |  | Replicate 6 | | 101.35 | 1.69 | 0.58 | |  |  |  |  | Replicate 6 | | 26.40 | | | 1.34 | 0.23 | |  |
|  |  | **Average** | | **84.42 ± 57.96** | | |  | |  | |  | | **Average** | | **23.55 ± 19.40** | | | |  | |

**Supplementary Table S2** - RNA recovery rate achieved after RNA extraction and cDNA synthesis processes for *P. aeruginosa* planktonic samples. For each bacterial concentration five technical replicates (S1 to S5 for 10^9^ CFU/mL; S6 to S10 for 10^5^ CFU/mL) were used.

| **Sample** | | ***ref* mRNA (transcripts/μL)** | **Recovery Rate (%)** | **Sample** | | ***ref* mRNA (transcripts/μL)** | **Recovery Rate (%)** |
| --- | --- | --- | --- | --- | --- | --- | --- |
| High-bacterial biomass (1×10^9^ CFU/mL) |  | 1×10^11^ | 3.75 | Low-bacterial  biomass  (1×10^5^ CFU/mL) |  | 1×10^11^ | 0.42 |
|  |  | 1×10^10^ | 1.99 |  |  | 1×10^10^ | 0.54 |
|  |  | 1×10^9^ | 3.87 |  |  | 1×10^9^ | 0.27 |
|  |  | 1×10^8^ | 12.70 |  |  | 1×10^8^ | 5.82 |
|  |  | 1×10^7^ | 19.7 |  |  | 1×10^7^ | 2.25 |

**Supplementary Table S3.** Determination of the experimental PA/SA ratio, in three artificial dual-species planktonic consortia, based on the normalized gene expression of the 16S rRNA gene to the exogenous *ref* mRNA control.

| **Ratio (PA/SA)** | **Ct**  ***ref* mRNA** | **Ct**  ***16S rRNA* (PA)** | **Ct**  ***16S rRNA* (SA)** | **Normalized expression – *ref* mRNA/*16S rRNA* (PA)** | **Normalized expression – *ref* mRNA /*16S rRNA* (SA)** | **Normalized expression - *16S rRNA* (PA)/*16S rRNA* (SA)** | **Experimental Ratio (PA/SA)** |
| --- | --- | --- | --- | --- | --- | --- | --- |
| **10** | 30.81 ± 0.22 | 17.26 ± 0.10 | 16.75 ± 0.12 | 12025.94 ± 1043.12 | 17191.07 ± 1568.97 | - | **12.86 ± 1.12** |
| **1** | 30.15 ± 0.10 | 19.43 ± 0.22 | 15.52 ± 0.25 | 1722.95 ± 353.52 | 25903.89 ± 5794.12 | 18.17 ± 0.70 | **1.22 ± 0.25** |
| **0.1** | 30.8 ± 0.69 | 22.42 ± 0.75 | 15.26 ± 0.68 | 332.88 ± 27.39 | 47576.43 ± 1392.88 | - | **0.13 ± 0.01** |

**Supplementary Table S4.** Relative quantification of *P. aeruginosa*-*S. aureus* dual-species biofilms using qPCR.

| **Sample** | **Ct**  **Luciferase** | **Ct**  **16S rRNA (PA)** | **Ct**  **16S rRNA (SA)** | **Normalized expression - Luciferase/16S rRNA (PA)** | **Normalized expression - Luciferase/16S rRNA (SA)** | **Normalized expression - 16S rRNA (PA)/**  **16S rRNA (SA)** | **qPCR Ratio (PA/SA) (Log_10_)** |
| --- | --- | --- | --- | --- | --- | --- | --- |
| **PA+SA 24 h** | 33.80 ± 0.46 | 12.06 ± 0.37 | 18.13 ± 1.06 | 3550489.81 ± 457976.33 | 59823.47 ± 27499.13 | 18.17 ± 0.70 | **3.09 ± 0.26** |
| **PA+SA 48 h** | 34.02 ± 0.41 | 13.63 ± 0.30 | 22.35 ± 0.64 | 1437789.56 ± 449510.04 | 3555.37 ± 1434.45 | 18.17 ± 0.70 | **3.88 ± 0.28** |

**Supplementary Table S5**- Variability in the quantification of *P. aeruginosa-S. aureus* dual-species biofilms by plate count (CFU/mL) and flow cytometry (Counts/mL).

| **Sample** | **Species** | **CFU/mL** | **Δ Log_10_ (CFU/mL)** | | **Counts/mL** | | **Δ Log_10_ (Counts/mL)** | |
| --- | --- | --- | --- | --- | --- | --- | --- | --- |
| **PA+SA 24 h** | *P. aeruginosa* | 3.24×10^8^ ± 1.33×10^8^ | 2.78 ± 0.50 | | 5.04×10^7^ ± 2.93×10^7^ | |  | |
|  | *S. aureus* | 8.60×10^5^ ± 9.00×10^5^ |  |  | 2.64×10^6^ ± 2.48×10^6^ | | 1.36 ± 0.19 | |
| **PA+SA 48 h** | *P. aeruginosa* | 3.51×10^8^ ± 9.47×10^7^ | 7.02 ± 0.62 | 1.27×10^8^ ± 1.78×10^7^ | | 2.35 ± 0.33 | |  |
|  | *S. aureus* | 9.10×10^1^ ± 1.07×10^2^ |  | 6.89×10^5^ ± 4.82×10^5^ | |  |  |  |

**Supplementary Table S6** - Oligonucleotide sequences of the primers used for gene expression quantification by qPCR and target gene function.

| **Organisms** | | **Primer** | | **Forward** | **Reverse** | **Primer Efficiency** | **R2** | **Melting Temperature (ºC)** | **Amplicon size (bp)** | **Gene Function** |
| --- | --- | --- | --- | --- | --- | --- | --- | --- | --- | --- |
| *S. aureus* | 16S | | GGTCTTGCTGTCACTTATAGATGG | | CGGAAGATTCCCTACTGCTG | 90.4 | 0.996 | 59.2/59.8 | 164 | Housekeeping |
| *S. aureus* | *sodA* | | ATGGCGGTGGTCATTTTAAC | | ACCAAGTCCAACCTGATCCA | 110.0 | 0.993 | 59.7/60.4 | 171 | Stress response (Superoxide dismutase) |
| *S. aureus* | *sarA* | | TGTTTGCTTCAGTGATTCGTTT | | CATCAGCGAAAACAAAGAGAAA | 94 | 0.997 | 59.8/59.5 | 233 | Virulence Regulator/ Quorum sensing |
| *S. aureus* | *agrB* | | GCACATGCACCTTCATCTTTT | | CCGGGATAGGCTTCTTCTTAG | 108.6 | 0.969 | 60.1/59.4 | 178 | Quorum sensing |
| *S. aureus* | *hld* | | TTTTTAGTGAATTTGTTCACTGTGTC | | TAATTAAGGAAGGAGTGATTTCAATG | 103.3 | 0.995 | 58.8/59.1 | 100 | Delta-hemolysin |
| *P. aeruginosa* | 16S | | GGAGAAAGTGGGGGATCTTC | | CCGGTGCTTATTCTGTTGGT | 90.6 | 0.997 | 59.9/59.9 | 316 | Housekeeping |
| *P. aeruginosa* | *rhlA* | | GCGCGAAAGTCTGTTGGTAT | | ATTTCCACCTCGTCGTCCTT | 90.9 | 0.995 | 60.3/60.9 | 249 | Rhamnolipids |
| *P. aeruginosa* | *pqsA* | | ACCGCGAAGGACACACTATC | | GGCAGGTAGGAACCAGAACC | 90.5 | 0.995 | 60.1/60.9 | 297 | HQNO |
| *P. aeruginosa* | *mucA* | | CTGGACGAGGAGTTGGTGAT | | GCGTCTGTACAACCAGAACG | 88.0 | 0.992 | 60.1/59.4 | 153 | Alginate Overexpression |
| - | Luciferase | | TACAACACCCCAACATCTTCGA | | GGAAGTTCACCGGCGTCAT | 108.6 | 0.990 | 59.6/60.0 | 67 | reference mRNA |
